# Potential application of serological tests on fluids from carcasses: detection of antibodies against *Toxoplasma gondii *and *Sarcoptes scabiei *in red foxes (*Vulpes vulpes*)

**DOI:** 10.1186/1751-0147-54-13

**Published:** 2012-03-01

**Authors:** Eva-Britt Jakubek, Roland Mattsson, Torsten Mörner, Jens G Mattsson, Dolores Gavier-Widén

**Affiliations:** 1 Department of Virology, Immunobiology and Parasitology, National Veterinary Institute, SE-75189 Uppsala, Sweden; 2 Department of Pathology and Wildlife Diseases, National Veterinary Institute, SE-75189 Uppsala, Sweden; 3 Department of Biomedical Sciences and Veterinary Public Health, University of Agricultural Sciences (SLU), SE-75189 Uppsala, Sweden; 4 Department of Medical Epidemiology and Biostatistics, Karolinska Institutet, SE-17177 Stockholm, Sweden

## Abstract

**Background:**

Serological surveys for disease investigation of wild animal populations require obtaining blood samples for analysis, which has logistic, ethic and economic difficulties. Applying serological test to fluids collected from dead animals is an alternative. The aim of this study was to assess if antibodies could be detected in two types of fluids collected from 56 carcasses of red foxes (*Vulpes vulpes*): pleural fluid and lung extract.

**Findings:**

In 22 (39%) foxes antibodies against *Sarcoptes scabiei *were detected in both fluid types by ELISA and Western blot. In 46 (82%) foxes, antibodies against *Toxoplasma gondii *were detected in pleural fluid and in 41 (73%) in lung extract applying a Toxo-screen test (DAT). Antibodies were still detectable in the same fluids kept at room temperature for 28 days, although in fewer foxes (16 and 14 foxes tested for *T. gondii *in lung extract and pleural fluid respectively; and 1 and 4 tested for *S. scabiei *in lung extract and pleural fluid respectively.

**Conclusions:**

These results indicate the potential utility of using fluids from carcasses for antibody screening of wild animals at the population level.

## Findings

Serological surveys are widely applied to study the presence and distribution of infectious diseases in wild animal populations. They are most often conducted as active surveillance programs on blood samples obtained from hunted animals, which implies restrictions with regards to the animal species, time of the year, age category and geographical distribution that can be tested. Serological testing of diseased wild animals necessitates immobilization or euthanasia. An advantageous alternative is to conduct antibody tests on body fluids obtained from carcasses. Few studies have investigated the presence of antibodies in fluids from carcasses in wild animals in Europe [1-3]. A main limitation of testing fluids from carcasses is its decay. The aim of the present study was to obtain a preliminary indication of the utility of conducting antibody detection tests on 2 types of fluids collected from carcasses of red foxes from which no serum was available, and to obtain an indication of the stability and persistence of antibodies in the fluids subjected to storing at room temperature (RT) for up to 28 days. The study investigated antibodies against two parasites that frequently infect red foxes in Sweden, the mite *Sarcoptes scabiei *which causes sarcoptic mange and the obligate intracellular protozoan *Toxoplasma gondii *which causes toxoplasmosis, a zoonotic infection [4,5]. Fifty-six carcasses of red foxes from various parts of Sweden, culled due to suspicion of mange, were submitted to the National Veterinary Institute (SVA), Uppsala, Sweden, for necropsy, in years 2005 and 2006 for a mange-targeted investigation as part of the wildlife disease surveillance program. The foxes were 37 males, 17 females and 2 with no record of sex. Their age, as estimated by dentition, varied between yearlings and animals older than 5 years. The body weight ranged between 3.3 and 10.0 kg with a mean weight of 5.4 kg. The skin and fur was inspected for signs indicative of sarcoptic mange [4]. The time between death and post mortem examination was unknown. From each fox, a piece of about 5 cm^3 ^from the apex of the left lung lobe was collected in an empty tube, and fluid from the thoracic cavity was sampled in another tube. The lung sample was cut into pieces of approximately 1 cm^3 ^and placed in 5 individual tubes containing 1 ml phosphate buffered saline (PBS), pH 7.2. The fluid from the thoracic cavity was divided into 5 portions. On the day of necropsy (day 0), a tube with lung was left at RT (20-22°C) for 20 min, then agitated for 2 min and centrifuged at 800 *g *for 10 min, as previously described [6]. The supernatant was collected and stored at -20°C until tested. A tube with cavity fluid was centrifuged and the supernatant was stored in the same way. The remaining 4 tubes were kept at room temperature for 7, 14, 21, and 28 days, respectively. On these days the samples were treated and stored as described for day 0. For detection of antibodies to *T. gondii *a commercial direct agglutination test (DAT), Toxo-Screen (bioMérieux, Lyon, France) was used according to the manufacturer's instructions and including the positive and negative controls in the kit. The samples were screened in duplicates at the dilutions 1:40 and 1:4000. Sera collected from a red fox 1 month after intravenous inoculation with 10^5 ^*T.gondii *(RH isolate) tachyzoites was used as an additional positive control. Serum from an uninfected fox was used as an additional negative control.

Antibodies against *S. scabiei *were assayed by an indirect ELISA method, at the same laboratory and under the same conditions as previously described [4]. The method has been evaluated for detection of antibodies against *S. scabiei *in red foxes in Sweden [4] and the same cut off OD values were used. Briefly, the method uses monoclonal mouse antidog-IgG which recognizes fox antibodies bound to the antigen on the plate, and rabbit antimouse Ig labelled with horse-radish peroxidise [4]. Known positive and negative sera were used as controls [7]. The conjugate was a mixture of monoclonal antibodies directed to dog IgG (National Veterinary Institute, Uppsala, Sweden) and rabbit-anti-mouse antibodies conjugated with horse radish peroxidase (Dako AS, Copenhagen, Denmark). The samples were diluted 1:100 and tested in duplicates. Samples with an OD value more than 0.165 were further tested with western blot (WB). The control sera and the secondary antibodies were the same as for *S. scabiei *ELISA. The signal was visualized with BCIP/NBT (Sigma), and pre-stained molecular weight markers (New England Biolabs, MA) were used to estimate the molecular weights. A serum deemed *S. scabiei *antibody positive if reactions to at least 3 of the immunodominant antigens were recorded [7].

The result from the Toxo-screen showed that 40 (71%) of the 56 foxes (day 0) had an antibody titer equal or greater than 1:40 in both pleural fluid and in lung extract and were regarded as positive. Further, six pleural fluid samples were positive in the test while the corresponding lung samples were negative. One lung sample was positive while pleural fluid was negative. The remaining 9 samples were negative in both pleural fluid and lung extract.

**Figure 1:**
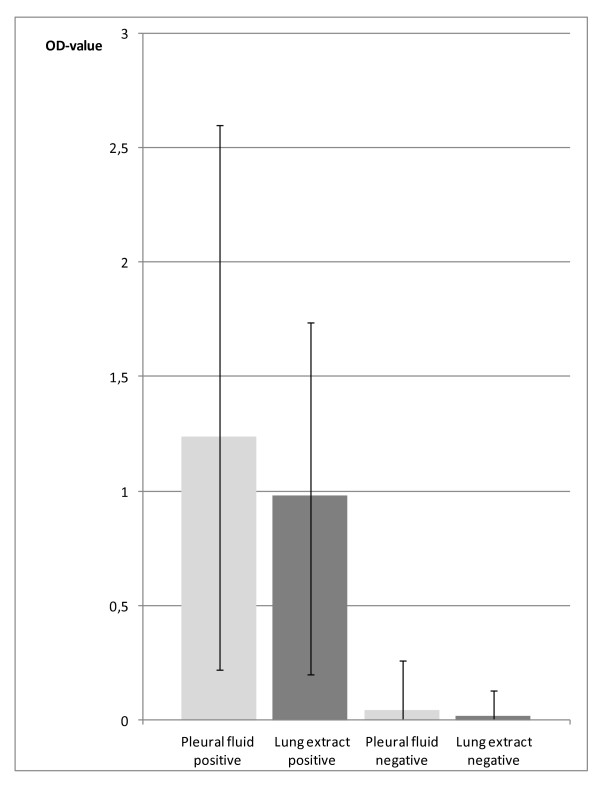
**The IgG antibody response against Sarcoptes scabiei in pleural fluid and lung extract from 57 carcasses of red foxes, analysed by ELISA**.

**Figure 2:**
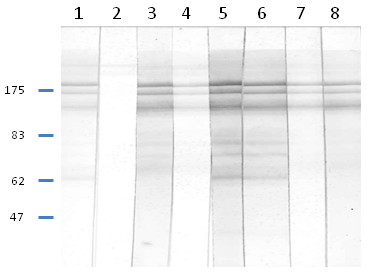
**Immunoblot analysis of *Sarcoptes scabiei *antibody responses under reducing conditions**. Strips were incubated with sera from a positive dog (1), a negative dog (2), pleural fluids from foxes with OD value over 0.165 in ELISA (3, 5, 7), lung extracts from foxes with OD value over 0.165 in ELISA (4, 6, 8).

A total of 22 (39%) of the 56 foxes showed gross skin lesions indicative of sarcoptic mange, in one fox the presence of mange lesions was considered inconclusive and the remaining animals (33 foxes) showed no typical mange lesions. In the *Sarcoptes *ELISA, all samples from the 22 foxes with mange lesions, had an OD value over 0.165 (the average OD value for the positive control was 1.4 and for the negative control 0.154). The animal that was inconclusive for mange at necropsy and 32 out of the 33 foxes with no macroscopic skin lesions had OD values below 0.020. However, a single animal with no mange lesions had an OD value of 0.260 in the pleural fluid sample and 0.130 in the lung extract (Figure 1). All the fluid samples from the 22 foxes with mange skin lesions were also analyzed by WB. Twenty of these foxes showed banding patterns typical for *S. scabiei *antibodies in both fluid samples (Figure 2). One fox showed no reactions in the WB of any of the samples (the samples were reanalyzed with the same result) and another fox was positive in the ELISA but only the lung extract sample yielded typical banding patterns in WB. This sample was from day 0. The pleural fluid from that day did not show any typical banding pattern, while the samples tested after 1, 2 and 3 weeks of storage gave typical banding patterns for an animal infected with *S. scabiei*. The negative result at day 0 might be a false negative and remains unexplained.

The correlation between positivity and host factors, such as gender, age, weight, and geographic origin, and degree of autolysis was not analysed because of the small numbers of animals.

**Figure 3:**
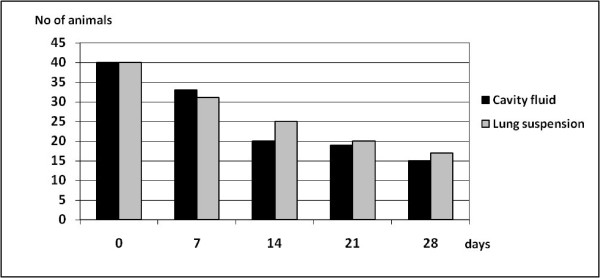
**The influence of sample storage time on the number of animals identified as antibody positive for *Toxoplasma gondii *as demonstrated by Toxo-screen**. Antibody titers were analyzed in thoracic cavity fluid and in lung suspension/extract samples stored at room temperature for up to 4 weeks.

**Figure 4:**
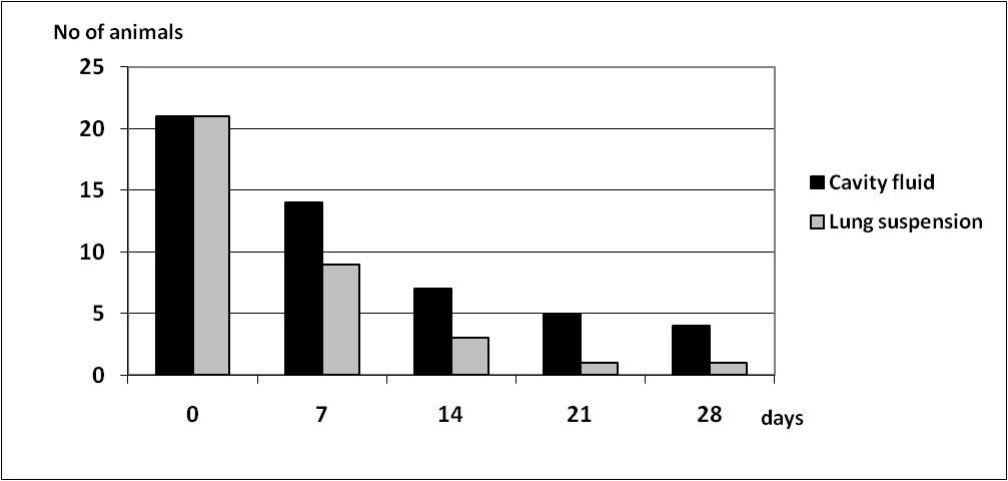
**The influence of sample storage time on the number of animals identified as antibody positive for *Sarcoptes scabiei *as demonstrated by ELISA**. Antibody levels were analyzed in thoracic cavity fluid and in lung suspension/extract samples stored at room temperature for up to 4 weeks.

Persistence of antibodies in body fluid samples stored at room temperature was investigated. In samples from the start of the experiment (day 0), 40 of the 56 foxes had a titer of 1:40 or higher in the Toxo-screen DAT assay in both pleural fluid and lung extract. At the end of the experiment, day 28, 16 of the lung extract samples were still positive while 14 of the 46 cavity fluid samples remained positive (Figure 3). At the day of autopsy (day 0), 22 of the 56 fox samples had an OD value much greater than the negative control in the *Sarcoptes *ELISA for both pleural fluid and lung extract. At the end of the 28-days-long experiment all but one of the lung extract samples were recorded as negative while 4 of the 23 pleural fluid samples still remained positive (Figure 4). The antibodies appeared to degrade faster in lung extract than in pleural fluid when tested with the *Sarcoptes *ELISA, while the contrary was observed when tested with the Toxo-screen assay from day 14 and subsequently. Both types of samples were obtained during necropsy under clean conditions and the reasons for the differences could not be established.

Serological assays are useful diagnostic tools. However, availability of serum samples from wild animals is often limited. Carcasses with various degrees of decomposition are often the only material available. A number of reports describe the detection of antibodies in other types of body fluids which can serve as alternative material to serum [8-11].

In this study the presence of antibodies in fluids from thoracic cavity and in lung extract were selected for evaluation because these samples can be easily obtained and stored, and lung is often included in frozen tissues archives. Storing fluid samples under suboptimal conditions (room temperature) partly simulated the decay of material in the field, but did not completely represent all factors affecting the decomposition. Investigating the persistence of antibodies in such samples provided however, presumtive information about the potential utility of such alternative samples.

In order to follow the standard protocols of the antibody detection methods applied in this study, the fluid samples were diluted as if they had been serum samples. The antibody levels in fluid samples are lower than those in serum, and the further dilutions have likely resulted on an underestimate of the true antibody prevalence and/or levels.

Antibodies against *S. scabiei *analysed by ELISA were detected in the pleural fluid of all the foxes with skin lesions of mange and in 95% of the foxes (21 of 22 foxes) in their lung extract. However, one fox with no mange lesions showed antibodies in pleural fluid but not in the lung extract and both samples were negative at the WB test. It is possible that this fox had a mild form of mange with grossly undetectable lesions and low antibody levels.

The analysis for toxoplasma showed that 71% of the foxes had detectable antibodies in the two sample types. No other methods were applied to confirm toxoplasmosis in the foxes. However, the high prevalence is in agreement with a number of similar studies on foxes from other European countries, i.e. UK (20%), Belgium (98.4%), and Hungary (68%) [11-13]. In an earlier antibody study on healthy Swedish foxes collected between 1991 and 1999, 38% of 221 red foxes had antibodies to *T. gondii *applying the same method and conducted at the same laboratorium as in this study [5]. The reasons for higher prevalence in the present study cannot be clearly elucidated, one factor could be the different years of sampling.

A previous study showed that IgG antibodies may be remarkably stable in decomposed lung tissue samples which had been stored for 48 days and were overgrown with bacteria and fungi [14]. In the present study it was found that the antibody levels in fluids collected from carcasses started to decline after the first week of incubation at room temperature, and the number of positive foxes decreased further in subsequent weeks. However, some of the samples (up to 39%, 16/41) were still positive at day 28. This observation supports the findings of a previous study that showed the stability of antibodies in carcasses over 11 days [15] but also shows that the number of false negative foxes increased with storage time, likely due to the decay of the antibodies.

Even though this study did not investigate the true length of the persistence of antibodies in carcasses and the sensitivity of the antibody detection methods applied to fluids in comparison to serum, the results show that antibodies could be detected in a high proportion of the samples. Based on these results, further studies may be justified to investigate the sensitivity of detection. It is expected that a significant proportion of the fluid samples may give false negative results, limiting the usefulness of testing fluids to assess the infectious status of individual animals, or at least diminishing the reliability of a negative result. However, testing of fluids as an alternative to serum may be conducted with advantage for antibody screening of populations, at least for infections of high prevalence, once the performance of the particular method on specific well-characterized alternative fluid samples is evaluated. Nonetheless, it is expected that the antibody prevalence testing fluid samples will be underestimated and lower than that testing serum samples. Additionally, decay of carcasses will further decrease the sensitivity of antibody detection. Other studies in wildlife have shown that even though Brucella-antibody levels in lung extracts of chamois (*Rupicapra rupicapra*) were one-to three folds lower than in serum, using lung allowed testing of a much larger sample size and was useful in monitoring the infection. In the present study we intended to obtain preliminary information on the utility of antibody detection for certain infections done in fluids alternative to serum with the vision of using a large frozen tissue and fluids bank of wildlife samples collected during the last 25 years at the National Veterinary Institute (SVA), Uppsala, Sweden, in the context of the EU project WildTech "Novel Technologies for Surveillance of Emerging and Re-emerging infections of Wildlife".

## Competing interests

The authors declare that they have no competing interests.

## Authors' contributions

EBJ conducted the laboratory analyses and drafted the manuscript. RM worked on the sample collection, preparation and storage and gathering of the animal data. DGW contributed to the analysis and presentation of the data and writing of the manuscript, TM contributed to the design of the study and writing of the manuscript. JGM contributed to the design of the study, analysis and presentation of the data and writing of the manuscript. All authors read and approved the final manuscript.

## References

[B1] Wolfe A Hogan S Maguire D Fitzpatrick C Vaughan L Wall D Red foxes (*Vulpes vulpes*) in Ireland as hosts for parasites of potential zoonotic and veterinary significance Vet Rec 2001 149 759 763 11808662

[B2] Almería S Ferrer D Pabón M Castellá J Manas S Red foxes (*Vulpes vulpes*) are a natural intermediate host of *Neospora caninum* Vet Parasitol 2002 107 287 294 10.1016/S0304-4017(02)00162-0 12163240

[B3] Ryser-Degiorgis MP Jakubek EB Hård af Segerstad C Bröjer C Mörner T Jansson DS Lundén A Uggla A Serological survey of *Toxoplasma gondii *infection in free-ranging Eurasian Lynx (Lynx lynx) from Sweden J Wildl Dis 2006 42 1 182 187 16699164 10.7589/0090-3558-42.1.182

[B4] Bornstein S Frössling J Näslund K Zakrisson G Mörner T Evaluation of a serological test (indirect ELISA) for the diagnosis of sarcoptic mange in red foxes (*Vulpes vulpes*) Vet Dermatol 2006 17 411 416 10.1111/j.1365-3164.2006.00548.x 17083572

[B5] Jakubek EB Bröjer C Regnersen C Uggla A Seroprevalence of *Toxoplasma gondii *and *Neospora caninum *in Swedish red foxes (Vulpes vulpes) Vet Parasitol 2001 102 167 172 10.1016/S0304-4017(01)00513-1 11705663

[B6] Mörner T Sandström G Mattsson R Comparison of serum and lung extracts for surveys of wild animals for antibodies to *Francisella tularensis *biovar palaearctica J Wildl Dis 1988 24 10 14 3280838 10.7589/0090-3558-24.1.10

[B7] Bornstein S Thebo P Zakrisson G Evaluation of an enzyme-liked immunosorbent assay (ELISA) for the serological diagnosis of canine sarcoptic mange Vet Dermatol 1996 7 21 28 10.1111/j.1365-3164.1996.tb00222.x 34644993

[B8] Gorin AB Stewart P Gould J Concentrations of immunoglobulin classes in subcompartments of the sheep lung Res Vet Sci 1979 26 126 128 472485

[B9] Lundén A Lind P Olsson-Engvall E Gustavsson K Uggla A Vågsholm I Serological survey of *Toxoplasma gondii *infection in pigs slaughtered in Sweden Scand J Infect Dis 2002 34 362 365 10.1080/00365540110080205 12069021

[B10] Hamilton CH Gray R Wright SE Gangadharan B Laurenson K Innes EA Prevalence of antibodies to *Toxoplasma gondii *and *Neospora caninum *in red foxes (*Vulpes vulpes*) from around the UK Vet Parasitol 2005 130 169 173 10.1016/j.vetpar.2005.03.020 15893084

[B11] Prestud KW Åsbakk K Fuglei E Mørk T Stien A Ropstad E Tryland M Gabrielsen GW Lydersen C Kovacs KM Loonen MJJE Sagerup K Oksanen A Serosurvey for Toxoplasma gondii in arctic foxes and possible sources of infection in the high Arctic of Svalbard Vet Parasitol 2007 150 6 12 10.1016/j.vetpar.2007.09.006 17950534

[B12] Buxton D Maley SW Pastoret P Brochier B Innes EA Examination of red foxes (*Vulpes vulpes*) from Belgium for antibody to *Neospora caninum *and *Toxoplasma gondii* Vet Rec 1997 141 308 309 10.1136/vr.141.12.308 9330477

[B13] Jakubek EB Farkas R Pálfi V Mattsson JG Prevalence of antibodies against *Toxoplasma gondii *and *Neospora caninum *in Hungarian red foxes (*Vulpes vulpes*) Vet Parasitol 2007 144 39 44 10.1016/j.vetpar.2006.09.011 17045742

[B14] Jakubek EB Uggla A Persistence of *Neospora caninum*-specific immunoglobulin G antibodies in bovine blood and lung tissue stored at room temperature J Vet Diagn Invest 2005 17 458 460 10.1177/104063870501700508 16312237

[B15] Tryland M Handeland K Bratberg AM Solbakk I-T Oksanen A Persistence of antibodies in blood and body fluid in decaying fox carcasses, as exemplified by antibodies against *Microsporum canis* Acta Vet Scand 2006 48 1 4 10.1186/1751-0147-48-10 PMC1553463 16987389

